# Developing a preoperative serum metabolome-based recurrence-predicting nomogram for patients with resected pancreatic ductal adenocarcinoma

**DOI:** 10.1038/s41598-019-55016-x

**Published:** 2019-12-09

**Authors:** Seoung Yoon Rho, Sang-Guk Lee, Minsu Park, Jinae Lee, Sung Hwan Lee, Ho Kyoung Hwang, Min Jung Lee, Young-Ki Paik, Woo Jung Lee, Chang Moo Kang

**Affiliations:** 1 Division of Hepatobiliary and Pancreatic Surgery, Department of Surgery, Yonsei University College of Medicine, Seoul, Korea; 2 Yonsei Pancreatobiliary Cancer Center, Severance Hospital, Seoul, Korea; 3 Department of Laboratory Medicine, Severance Hospital, Yonsei University College of Medicine, Seoul, Korea; 4 Biostatistics Collaboration Unit, Yonsei University College of Medicine, Seoul, Republic of Korea; 5 Department of Systems Biology, University of Texas MD Anderson Cancer Center, Houston, TX, USA; 6 Yonsei Proteome Research Center and ‡Department of Integrated OMICS for Biomedical Science and Department of Biochemistry, Yonsei University College of Life Science and Biotechnology, Seoul, Korea

**Keywords:** Cancer genomics, Surgical oncology

## Abstract

We investigated the potential application of preoperative serum metabolomes in predicting recurrence in patients with resected pancreatic cancer. From November 2012 to June 2014, patients who underwent potentially curative pancreatectomy for pancreatic ductal adenocarcinoma were examined. Among 57 patients, 32 were men; 42 had pancreatic head cancers. The 57 patients could be clearly categorized into two main clusters using 178 preoperative serum metabolomes. Patients within cluster 2 showed earlier tumor recurrence, compared with those within cluster 1 (p = 0.034). A nomogram was developed for predicting the probability of early disease-free survival in patients with resected pancreatic cancer. Preoperative cancer antigen (CA) 19–9 levels and serum metabolomes PC.aa.C38_4, PC.ae.C42_5, and PC.ae.C38_6 were the most powerful preoperative clinical variables with which to predict 6-month and 1-year cancer recurrence-free survival after radical pancreatectomy, with a Harrell’s concordance index of 0.823 (95% CI: 0.750–0.891) and integrated area under the curve of 0.816 (95% CI: 0.736–0.893). Patients with resected pancreatic cancer could be categorized according to their different metabolomes to predict early cancer recurrence. Preoperative detectable parameters, serum CA 19–9, PC.aa.C38_4, PC.ae.C42_5, and PC.ae.C38_6 were the most powerful predictors of early recurrence of pancreatic cancer.

## Introduction

Pancreatic cancer is one of the most lethal cancers arising from the gastrointestinal tract. It is estimated that pancreatic cancer will become the second highest cause of cancer-related death by 2030^1–3^. Potentially curative pancreatectomy is regarded as the most effective monotherapy; however, only 15–20% of patients are candidates for radical operation at diagnosis. Most patients with resected pancreatic cancer experience cancer recurrence, especially to the liver, lung, and peritoneum. Thus, surgery followed by postoperative adjuvant chemotherapy is the standard of care, although this only provides long-term survival of less than 25–30%^4^.

Many studies have investigated the use of clinicopathological factors for predicting cancer recurrence in patients with resected pancreatic cancer and have suggested that lymph node metastasis^5^, perineural invasion^6,7^, lymphovascular invasion^8^, and incomplete resection^9^ are significantly associated with early tumor recurrence and poor survival outcomes after surgical intervention. However, having preoperatively detectable parameters with which to predict early cancer recurrence in resected pancreatic cancer would be more useful for patients, surgeons, and medical oncologists, helping them to decide whether to conduct surgical resection and consider the potential postoperative morbidity and mortality following radical pancreatectomy.

Comprehensive investigation using genomics, transcriptomics, proteomics, and metabolomics is essential to understanding cancer biology. The metabolome represents the final stage in the “omics” cascade and is thought to be the closest phenotype to the biological behavior of cancer^10^. The metabolomes of cancer patients are affected not only by endogenous expression of the cancer itself but also by exogenous factors related to the cancer, such as the environment and diet. Therefore, investigating cancer metabolomes can be a useful approach for discovering effective biomarkers^11,12^ Research in recent years has shown that metabolic reprogramming is one of the hallmarks of cancerous cells^13^ and metabolomic signatures have already been identified in pancreatic cancer, suggesting a potential application in personalized therapy for pancreatic cancer^14,15^ by allowing earlier and more precise diagnostics, prognostics, and prediction of new therapeutic targets. However, recent studies have almost entirely focused on the early detection of pancreatic cancer, with only a few studies reporting the long-term prognostic role of metabolomes in pancreatic cancers^16^.

In this study, we investigated the potential clinical application of preoperative serum metabolomes in predicting cancer recurrence in patients with resected pancreatic cancer. Furthermore, we intended to develop a preoperative serum metabolome-based nomogram with which to predict early recurrence of resected pancreatic cancer.

## Materials and Methods

### Patient data

From November 2012 to June 2014, among patients who underwent potentially curative pancreatectomy for pancreatic ductal adenocarcinoma, those with available preoperative blood samples and long-term follow up data were enrolled in this study. Medical records of the patients were retrospectively reviewed. Perioperative clinicopathological characteristics, such as age, sex, neoadjuvant treatment, jaundice, preoperative laboratory findings including cancer antigen (CA) 19–9, tumor size, tumor location, operative procedure, pathological findings, American Joint Committee on Cancer (AJCC) cancer stage, and postoperative adjuvant chemotherapy, were investigated. All laboratory variables (glucose level, total bilirubin, serum protein, albumin, CA 19–9 level) were investigated preoperatively at least 1 week before surgery. Among them, glucose level was checked after 8 hours of fasting before surgery.

### Ethical issues

This study protocol was approved by the institutional review board of Severance Hospital (IRB No. 4-2017-0503). The need to obtain informed consent was waived, because the serum metabolomes used in this retrospective study were collected from patient blood stored in a tissue bank prior to surgery. Curative intended pancreatectomy for pancreatic duct adenocarcinoma was performed according to standard criteria for patient selection and surgical procedures that have obtained international consensus. In addition, the methods used for the analysis of metabolomes are also widely used internationally. All methods were performed in accordance with the relevant guideline and regulation.

### Detecting preoperative serum metabolomes

In total, 188 metabolites were analyzed using a targeted metabolomics approach and Absolute IDQTM p180 kits (Biocrates Life Sciences AG, Innsbruck, Austria; Supplementary 1). The kit consists of a single sample preparation procedure, although two separate mass spectrometry (MS) analytical runs, a combination of liquid chromatography (LC) and flow-injection analysis (FIA) coupled to tandem mass spectrometry (MS/MS), are conducted. The kit enables simultaneous quantification of 21 amino acids, 21 biogenic amines, 40 acylcarnitines (Cx:y), 90 glycerophospholipids (14 lysophosphatidylcholines [lyso PCx:y] and 76 phosphatidylcholines [PC aa x:y or PC ae x:y]), 15 sphingolipids (SMx:y or SM [OH]x:y), and one hexose. Cx:y denotes the lipid side chain configuration, where x indicates the number of carbons in the side chain and y indicates the number of unsaturated chains. Of 188 metabolites analyzed, 42 metabolites were measured by LC-MS/MS and 146 metabolites by FIA-MS/MS. Amino acids and biogenic amines were analyzed quantitatively by LC-MS/MS using external calibration standards at seven different concentrations and isotope-labelled internal standards. The acylcarnitines, glycerophospholipids, sphingolipids, and sum of hexoses were measured by FIA-MS/MS using one-point internal calibration with representative internal standards. The results of lipids were classified as semi-quantitative since specific standards were not commercially available and accuracy could not be determined over a full quantification range.

For lipid nomenclature, each metabolome is described in accordance with the official lipid nomenclature provided by Lipid Maps. Also, annotations for the potential isomers of each metabolomes and the corresponding lipid map IDs are provided in Supplementary 2.

Serum samples were processed in strict accordance with the instructions provided by the manufacturer. After the addition of 10 µL of the supplied internal standard solution to each well of a 96-well extraction plate, 10 µL of each serum sample was added to the appropriate well. The plate was then dried under a gentle stream of nitrogen. The samples were derivatized with phenyl isothiocyanate and eluted with 5 mM ammonium acetate in methanol. Samples were diluted with either 40% methanol in water for LC-MS/MS analysis (15:1) or running buffer provided by the kit (Biocrates Solvent I) for FIA-MS/MS (20:1).

The LC‐MS/MS system comprised an Agilent 1290 Infinity HPLC system (Agilent Technologies Inc., Santa Clara, CA, USA) coupled to a QTRAP 5500 mass spectrometer (Sciex, Woodlands Central, Singapore) in the electrospray ionization mode. Amino acids and biogenic amines were analyzed via LC‐MS/MS in the positive mode. Five microliters of sample extract were injected onto an Agilent Zorbax Eclipse XDB C18 column (3.0 × 100 mm, 3.5 μm) protected by a SecurityGuard pre‐column (C18, 4 × 3 mm) (Phenomenex, Torrance, CA, USA) at 50 °C using a 9.5-min solvent gradient employing 0.2% formic acid in water (mobile phase A) and 0.2% formic acid in acetonitrile (mobile phase B). Twenty microliters of sample extract were used for FIA-MS/MS in the positive mode to measure acylcarnitines, glycerophospholipids, and sphingolipids, while hexoses were monitored in a subsequent run in the negative mode. All FIA injections were carried out using the mobile phase prepared by Biocrates Solvent I in an isocratic mode. The LC and MS settings for LC‐MS/MS and FIA-MS/MS mode are described in Supplementary 3. All metabolites were identified using multiple reaction monitoring as optimized and provided by Biocrates Life Sciences AG.

All measurements were made in a 96-well format. Analytical performance was monitored using three quality control (QC) samples (a low, medium, and high concentration). The three individual QC samples were placed at the beginning of an analytical run. Additional QC samples at the medium concentration were placed at the middle and end of each analytical run. Metabolite concentrations were calculated by employing a combination of Analyst^TM^ (Sciex) and MetIDQ^TM^ (Biocrates) software. We confirmed that the accuracy of QC samples was within the tolerance limit provided by the manufacturers and validated the data using MetIDQ^TM^ software before data processing. Because we tested all samples in a single well plate, we did not normalize the data to correct for batch effects.

Ten metabolites were not included for analysis because they were not detected in the majority of samples.

### Statistical analysis using hierarchical clustering

For exploration of high-throughput data, clustering and heatmap analysis were considered. Also, hierarchical clusters were used to generate models that could comprehensively consider independent variables, such as a Cox proportional hazards model. To convert highly correlated preoperative serum metabolomes into a grouped variable in a hierarchical way and to visualize how clusters formed, hierarchical cluster analysis considered the Euclidean distance as a distance measure and the ‘Ward.D2’ algorithm as a linkage method^17^.

### Determining the number of preoperative serum metabolome-based clustering groups

To properly determine the appropriate number of clustering groups, a hierarchical clustering algorithm based on the silhouette method was implemented in the R package *factoextra*.

### Other statistics and testing

Categorical variables are expressed as a frequency and percentage, and were analyzed by Fisher’s exact test. Continuous variables are described as a mean ± standard deviation when the normality assumption was satisfied and as a median (interquartile range) when it was not. When the normality assumption for continuous variables was violated, the Wilcoxon rank sum test was conducted instead of Student’s *t*-test.

### Adjusted p-value for multiple comparisons

P-values obtained from the comparison of two clusters were adjusted using the false discovery rate by the Benjamini-Hochberg procedure to counteract the problem of multiple comparisons. To appropriately reduce the number of variables used in the model, employing only larger differences between the clusters among the 178 metabolomes analyzed in this study, only variables with an adjusted p-value < 0.001 were used in the model.

### Building survival models and comparing the predictive power of recurrence-predicting models

Based on covariate variables, such as age, sex, neoadjuvant chemotherapy, tumor size, preoperative CA 19–9, jaundice, and tumor location, the significant prognostic factors for predicting 1-year disease-free survival were preferentially selected by univariate Cox proportional hazards models. Multivariate Cox proportional hazards models were used to construct a model that added not only prognostic factors but also the clustering groups and instrumental metabolomes that could distinguish between clusters. The best model including metabolomes was finally established with normalization of metabolomic data and the constraint that the last set of covariates must have a variation inflation factor <10. This was implemented in the R package *My.stepwise*. The proportionality assumption for the Cox model was also confirmed. Harrell’s concordance (C)-index and Heagerty’s integrated time-dependent area under the curve (iAUC) for each of the 1000 bootstrap samples used to assess model performance of established recurrence-predicting models^18,19^.

### Establishing nomogram

A nomogram for predicting the probability of 6- and 12-month disease-free survival for patients with resected pancreatic ductal adenocarcinoma was constructed on the basis of the Cox model that had the most predictive power among the considered models.

All statistical hypothesis tests were two-sided with a significance level of 0.05. All statistical analyses were implemented using R packages, version 3.4.0.

## Results

### General patient characteristics

All 57 patients were confirmed to have pancreatic ductal adenocarcinoma. In total, 32 patients (56.1%) were male and 25 were female, with an overall average age of 64.7 (±9.5) years. Pancreatic head cancers were found in 42 patients (73.7%) and pancreatic body and tail cancers in 15 patients (26.3%). Serum CA 19–9 at initial diagnosis was 1058.1 (U/mL) (±2474.4). Neoadjuvant chemoradiation therapy was provided for 12 patients (21.1%). Pancreaticoduodenectomy was performed in 41 patients, distal pancreatectomy with splenectomy in 15 patients, and total pancreatectomy in 1 patient. Resected tumor size was 2.8 cm (±1.2) cm in diameter, and the number of metastatic lymph nodes was 1.8 (±2.5).

### Preoperative serum metabolome-based clustering of patients with resected pancreatic cancer

**Figure 1:**
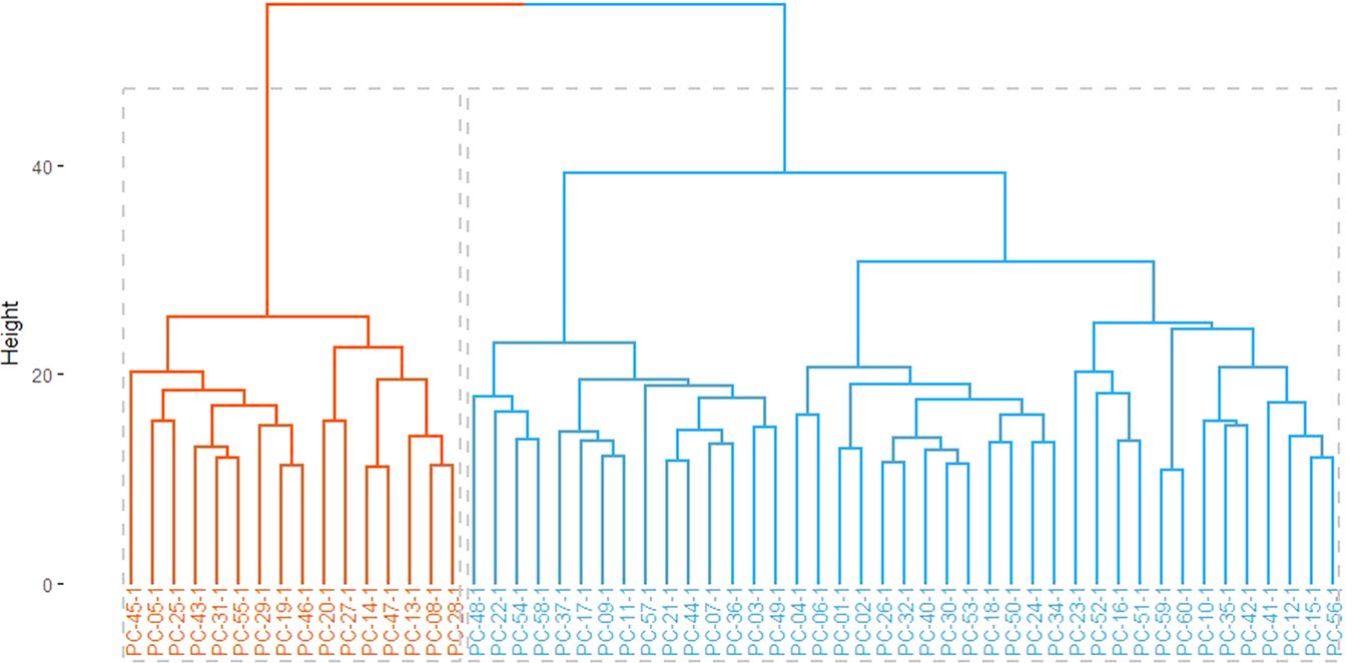
Cluster dendrogram according to the expression pattern of preoperative serum metabolomes.

The 57 patients could be clearly separated into two main clusters using 178 preoperative serum metabolomes. The hierarchical relationship among the resected pancreatic cancer patients with preoperative serum metabolomes is shown as a cluster dendrogram in Fig. 1.

Among the 178 detected metabolomes, the top 15 most differentiating metabolomes between two clustering groups are summarized in Table 1. Interestingly, all of the top 15 metabolomes were related to phosphatidylcholines (PC), which are differentiated according to the presence of an ester (“a”) or an ether (”e”) binding to the glycerol moiety. Among them, the “aa” (diacyl) form of PCs was found in 6 (40%) of the metabolomes, while 9 (60%) were related to the “ae” (acyl-alkyl) form of PCs. The value of the PC derivatives related to the top 15 preoperative serum metabolomes were found to be significantly lower in patients of cluster 2, compared with cluster 1.

**Table 1:** The 15 most significant metabolomes (adjusted p-value < 0.001) differentiating preoperative serum metabolome-based clustering groups (arranged in ascending order by adjusted p-values).

Metabolomes	Cluster 1 (N = 41)	Cluster 2 (N = 16)	p-value^†^	SMD^*^	Lower	Upper
PC.ae.C36_4	12054 (10989–12858)	7225 (6699.8–8850)	4.40E-05	2.357	1.624	3.076
PC.ae.C38_4	7136 (6450–8166)	5068.5 (4220–5402.8)	5.90E-05	2.057	1.357	2.744
PC.ae.C40_5	2574 (2302–3137)	1727 (1578.2–2070.8)	5.90E-05	1.983	1.291	2.662
PC.ae.C38_5	12576 (11306–13565)	8770.5 (7702.5–9768.2)	7.10E-05	2.143	1.434	2.839
PC.ae.C40_4	1386 (1238–1630)	957.5 (857–1120)	1.10E-04	1.989	1.296	2.669
PC.ae.C42_4	468 (435–543)	353 (318–411.8)	1.50E-04	1.748	1.079	2.405
PC.ae.C36_5	10046 (8675–10712)	6391.5 (5230.2–7379)	1.60E-04	2.028	1.331	2.712
PC.ae.C42_5	1181 (1049–1301)	872.5 (805.8–974)	2.10E-04	1.881	1.199	2.551
PC.ae.C44_4	235 (223–246)	184.5 (171.8–194.2)	2.50E-04	1.902	1.218	2.574
PC.aa.C40_6	41083 (34973–49833)	25533.5 (21715.2–32665)	3.30E-04	1.76	1.089	2.418
PC.aa.C40_4	1941 (1743–2226)	1314 (1134.2–1543.8)	4.30E-04	1.738	1.07	2.394
PC.ae.C38_6	7616 (6387–8615)	5007 (3841.8–,5751.2)	4.70E-04	1.8	1.126	2.462
PC.aa.C38_4	76637 (65875–87120)	54081 (44843.5–60592.8)	5.10E-04	1.781	1.108	2.441
PC.ae.C40_1	758 (667–905)	532 (450.8–586.5)	7.20E-04	1.669	1.007	2.319
PC.aa.C38_0	3047 (2659–3710)	2126.5 (1755–2487.2)	7.90E-04	1.685	1.021	2.337

^†^P-value from Wilcoxon rank sum test with false discovery rate by the Benjamini-Hochberg procedure.

*Standardized mean differences.

Cluster 1 is on the right (blue) and cluster 2 is on the left (red). The y-axis represents a measure of closeness of individual clusters.

### Clinicopathological differences between preoperative serum metabolome-based clustering groups

Comparing clinical and pathological characteristics between two preoperative metabolomic-based clustering groups, we found that patients in cluster 2 showed significantly higher preoperative serum glucose levels (122 [107, 180] vs. 180.5 [131.75, 324.5], p = 0.035). No other clinicopathological characteristics, including age, sex, CA 19–9, preoperative neoadjuvant treatment, tumor characteristics, laboratory, and pathological findings, were statistically significant between the two clusters (p > 0.05, Table 2).

**Table 2:** Clinicopathological characteristics according to preoperative serum metabolome-based clustering groups.

Demographics	Total (N = 57)	Cluster 1 (N = 41, 71.9%)	Cluster 2 (N = 16, 28.1%)	p-value	Effect size	95%CI Lower	95% CI Upper
**Age (years)**	67 (58–72)	67 (57–73)	67 (60.75–69.25)	0.742	0.014	−0.564	0.592
**Sex**				0.255	0.364	−0.219	0.944
1	25 (43.9)	20 (48.8)	5 (31.2)				
2	32 (56.1)	21 (51.2)	11 (68.8)				
**Neo-Tx**				0.287	0.096	−0.482	0.674
No	45 (78.9)	34 (82.9)	11 (68.8)				
Yes	12 (21.1)	7 (17.1)	5 (31.2)				
**Jaundice**				0.771	0.096	−0.482	0.674
No	34 (59.6)	25 (61)	9 (56.2)				
Yes	23 (40.4)	16 (39)	7 (43.8)				
**Glucose**	129 (110–206)	122 (107–180)	180.5 (131.75–324.5)	0.035	0.592	0.001	1.178
**Total bilirubin**	6.9 (6.4–7)	7 (6.4–7.1)	6.8 (6.38–7)	0.252	0.154	−0.425	0.732
Protein	4 (3.8–4.2)	4 (3.8–4.1)	4 (3.8–4.23)	0.703	0.099	−0.479	0.677
Albumin	1.2 (0.6–8.1)	1.2 (0.5–8)	1.25 (0.95–8.67)	0.742	0.189	−0.391	0.767
CA 19–9 (U/mL)	157 (32–555.1)	98.1 (26.2–524.3)	178 (49.6–625.33)	0.689	0.074	−0.504	0.652
**Tumor Size (cm)**	2.5 (2–3.3)	2.3 (2–3)	3 (2.25–3.85)	0.092	0.44	−0.145	1.022
Tumor location				>0.999	0.042	−0.536	0.62
Head	42 (73.7)	30 (73.2)	12 (75)				
Body + Tail	15 (26.3)	11 (26.8)	4 (25)				
**Operative procedure**				>0.999	0.235	−0.345	0.813
PD	3 (5.3)	2 (4.9)	1 (6.2)				
PPPD	38 (66.7)	27 (65.9)	11 (68.8)				
DPS	15 (26.3)	11 (26.8)	4 (25)				
TP	1 (1.8)	1 (2.4)	0 (0)				
**Differentiation**				0.662	0.216	−0.364	0.794
Well & Moderate	50 (89.3)	35 (87.5)	15 (93.8)				
Poor	6 (10.7)	5 (12.5)	1 (6.2)				
**Lymphatic Invasion**				0.752	0.201	−0.379	0.779
No	39 (68.4)	27 (65.9)	12 (75)				
Yes	18 (31.6)	14 (34.1)	4 (25)				
**Vascular Invasion**				0.236	0.406	−0.178	0.987
No	35 (61.4)	23 (56.1)	12 (75)				
Yes	22 (38.6)	18 (43.9)	4 (25)				
**Perineural Invasion**				0.74	0.317	−0.442	0.715
No	13 (22.8)	10 (24.4)	3 (18.8)				
Yes	44 (77.2)	31 (75.6)	13 (81.2)				
**Margin**				>0.999	0.044	−0.534	0.622
R0	47 (82.5)	34 (82.9)	13 (81.2)				
R1	10 (17.5)	7 (17.1)	3 (18.8)				
**AJCC 8**^**th**^ **T stage**				0.669	0.304	−0.278	0.883
T1	16 (28.1)	13 (31.7)	3 (18.8)				
T2	31 (54.4)	21 (51.2)	10 (62.5)				
T3	10 (17.5)	7 (17.1)	3(18.8)				
**AJCC 8**^**th**^ **N stage**				0.666	0.279	−0.302	0.858
N0	27 (47.4)	21 (51.2)	6 (37.5)				
N1	21 (36.8)	14 (34.1)	7 (43.8)				
N2	9 (15.8)	6 (14.6)	3 (18.8)				
**#Retrieved LNs**	16.93 ± 9.3	17.32 ± 9.86	15.9 ± 7.9	0.585	0.154	−0.425	0.732
**#Positive LNs**	1 (0–3)	0 (0–3)	1 (0–2)	0.858	0.114	−0.465	0.692
LNR	0.05 (0–0.15)	0 (0–0.15)	0.06 (0–0.13)	0.749	0.017	−0.561	0.595
**Postoperative adj-CTx**					0.129	−0.45	0.707
No	9 (15.7)	7 (17.0)	2 (12.5)				
Yes	48 (84.3)	34 (83.0)	14 (87.5)	0.720			

Abbreviations: Neo-Tx, neoadjuvant therapy; PD, pancreaticoduodenectomy; PPPD, pylorus-preserving pancreaticoduodenectomy; DPS, distal pancreatectomy with splenectomy; TP, total pancreatectomy; LN, lymph node; LNR, lymph node ratio; adj-CTx, adjuvant chemotherapy.

### Long-term oncological outcomes according to preoperative serum metabolome-based clustering groups

**Figure 2:**
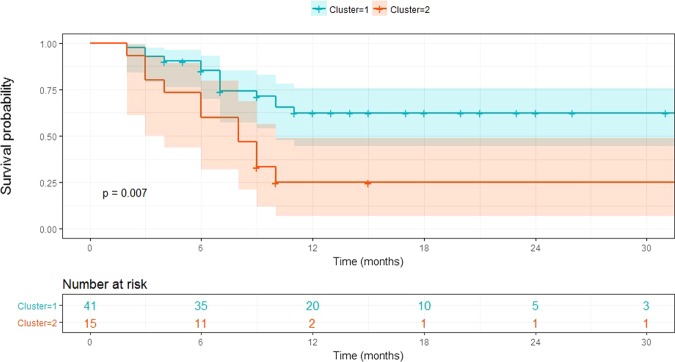
Disease-free survival according to preoperative serum metabolome-based clustering.

Disease-free survival differed significantly between the preoperative serum metabolome-based clustering groups. Patients within cluster 2 showed earlier tumor recurrence than those within cluster 1 (median 8 months [95% confidential interval (CI): 5.521–10.479] vs. median 15 months [95% CI: 8.375–21.625], p = 0.034). However, there was no statistical differences in terms of disease-specific survival between the two groups (p = 0.312) (Fig. 2).

### Determining factors to predict 1-year disease-free survival in resected pancreatic cancer

Among clinicopathological characteristics, preoperative serum metabolome-based cluster 2 (hazard ratio [HR] = 2.839, p = 0.015), tumor size (HR = 1.433, p = 0.015), and preoperative CA 19–9 (HR = 1.0001, p = 0.043) were identified as independent 1-year predicting factors after radical pancreatectomy for pancreatic ductal adenocarcinoma (Table 3). Other preoperatively detectable parameters, such as age (HR = 0.985, p = 0.483), sex (HR = 2.1774, p = 0.07), neoadjuvant chemotherapy (HR = 0.6097, p = 0.365), jaundice (HR = 1.3349, p = 0.474), and tumor location (HR = 0.5803, p = 0.277), failed to show prognostic significance in predicting 1-year disease-free survival.

**Table 3:** Predicting perioperative factors for estimating 1-year disease-free survival.

	Predicting 1-year disease-free survival			
	Univariate		Multivariate	
	HR (95% CI)	p-value	HR (95% CI)	p-value
Cluster 2 (ref. cluster 1)	2.874 (1.293–6.389)	0.01	2.839 (1.227–6.571)	0.015
Tumor Size	1.558 (1.18–2.057)	0.002	1.433 (1.073–1.912)	0.015
CA 19–9	1.0001 (1–1.0002)	0.054	1.0001 (1–1.0003)	0.043

### Developing recurrence-predicting nomogram using preoperative serum metabolomes in resected pancreatic cancer

Model performance was tested for accuracy in predicting the probability of early disease-free survival in resected pancreatic cancer (Table 4). We found that model 3, which considered preoperative CA 19–9 and three individual preoperative serum metabolomes (PC.aa.C38_4, PC.ae.C42_5, and PC.ae.C38_6), was the most powerful preoperative clinical model with which to predict 6-month or 1-year cancer recurrence-free survival after radical pancreatectomy, with a Harrell’s C-index of 0.823 (95% CI: 0.750–0.891) and an iAUC of 0.816 (95% CI: 0.736–0.893; Table 5).

**Table 4:** Clinically applicable model for predicting 1-year disease-free survival in resected pancreatic cancer.

	Model 1		Model 2		Model 3	
	HR (95% CI)	p-value	HR (95% CI)	p-value	HR (95% CI)	p-value
CA 19–9	1.0001 (1–1.0002)	0.054	1.0001 (1–1.0003)	0.018	1.0001 (0.9999–1.0002)	0.205
Cluster 2 (ref. cluster 1)			3.303 (1.443–7.556)	0.005		
PC.aa.C38_4					0.28 (0.134–0.587)	<0.001
PC.ae.C42_5					2.43 (1.245–4.743)	0.009
PC.ae.C38_6					0.558 (0.324–0.963)	0.036

Model 1: CA 19–9; model 2: model 1 + cluster 2; model 3: model 1 + metabolomes.

**Table 5:** Comparison of predictive power among three survival models according to C-index and iAUC.

	Harrell’s C-index (95% CI)	iAUC
Model 1	0.619 (0.487–0.733)	0.573 (0.507–0.653)
Model 2	0.695 (0.591–0.794)	0.684 (0.586–0.782)
Model 3	0.823 (0.75–0.891)	0.816 (0.736–0.893)
Model 1 vs. model 2	−0.076 (−0.209–0.021)	−0.111 (−0.207–−0.022)
Model 1 vs. model 3	−0.204 (−0.349–−0.08)	−0.243 (−0.346–−0.15)
Model 2 vs. model 3	−0.128 (−0.238–−0.04)	−0.132 (−0.227–−0.045)

**Figure 3:**
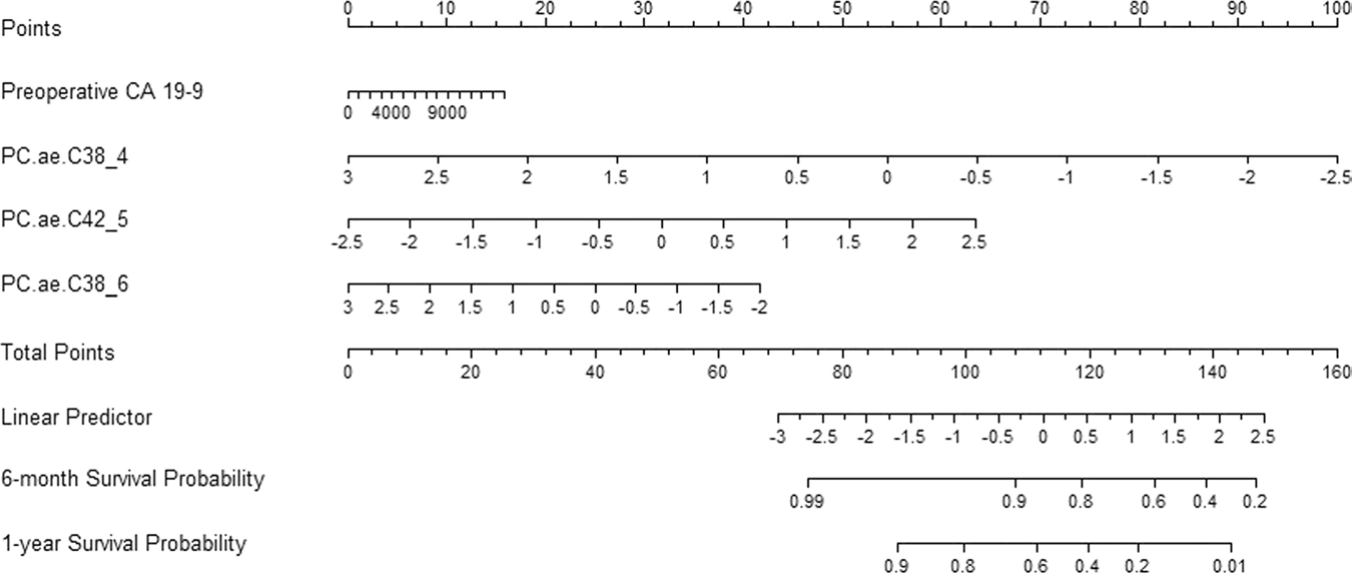
Nomogram for predicting 6-month and 1-year disease-free survival in patients with resected pancreatic cancer.

Setting aside tumor size, which must be determined by a pathologist during the postoperative period, a nomogram was developed considering only preoperative detectable parameters, such as preoperative CA 19–9 and three significant PC derivatives (Fig. 3).

## Discussion

Our present study demonstrated that patients with resected pancreatic cancer can be categorized into two groups according to their preoperative serum metabolomes. Of these two groups, one (cluster 2) was significantly associated with earlier cancer recurrence. This suggests the potential clinical application of preoperative serum metabolomes in elucidating the tumor biology of resected pancreatic cancer.

Interestingly, only preoperative serum glucose was significantly higher in patients of cluster 2, when comparing clinicopathological characteristics according to the clustering of different metabolomes. There are several studies reporting a potential association between serum glucose level and oncological outcomes in pancreatic cancer^20,21^. Raghavan *et al*.^22^ performed a comprehensive review to address the impact of diabetes on the prognosis of pancreatic cancer. Based on 38,777 patients from 31 studies, they found that diabetic patients with pancreatic cancer had significantly lower overall survival than those without diabetes (14.4 vs. 21.7 months; p < 0.001). Recently, Lv *et al*.^23^. also performed a meta-analysis to investigate the impact of diabetes on the clinical outcomes of resected pancreatic cancer. They found that new-onset diabetes conferred a negative impact on the survival of patients with resected pancreatic cancer. The observations that new-onset diabetes is strongly associated with pancreatic cancer^24^ and pancreatic resection in some cases can lead to improved serum glucose control suggest that pancreatic cancer may increase the level of serum glucose in patients and change their metabolomes. However, the underlying mechanism has not been elucidated yet. This potential correlation among serum glucose level, oncological outcome, and the metabolome is an interesting topic to be further investigated.

Most metabolomic studies have focused on the early diagnosis of pancreatic cancer^25–28^. Investigations of the potential relationship between metabolomes in pancreatic cancer patients and their prognoses are scarce. Fontana *et al*.^29^ developed a metabolites risk score (MRS) for predicting 1-year mortality risk in patients with pancreatic adenocarcinoma. They concluded that mass spectrometry-based metabolomic profiling of patients through their serum represented a valid tool for the identification of novel biomarkers with which to predict 1-year mortality risk in pancreatic cancer patients. However, the study population comprised only 27 patients, and most cases (74.1%) were reported to be unresectable. Considering that margin-negative resection is known to be the most effective treatment modality, the question of the potential role of MRS in this population is arising, because a small proportion of the patients who undergo resection will survive longer than those without surgical resection. Without analyzing the MRS, 1-year mortality is highly expected, because survival is estimated to be less than 1 year for patients with unresected pancreatic cancer^30,31^. Battini *et al*.^32^ also investigated the use of tumor metabolism profiling for predicting the clinical outcomes of pancreatic cancer patients. Although they suggested that metabolomic profiling based on 1 H high-resolution magic angle spinning nuclear magnetic resonance spectroscopy could provide important information for the characterization of pancreatic cancer and also predict long-term survival, they needed intact tissue obtained during surgical procedure for this analysis. Moreover, although they did not specify exact survival outcomes, the median survival was approximately 1 year when ethanolamine concentration was <0.740 nmol/mg. According to this scenario, questions remain regarding whether surgery should be performed for these patients. For a tailored surgical approach for resectable pancreatic cancer, it would be more helpful if surgeons, medical oncologists, and patients are able to obtain additional information on potential survival probability prior to surgical intervention. One study sought to predict survival outcomes through specific metabolites in serum. C. Yuan *et al*.^33^ investigated 82 metabolites in prediagnostic plasma by liquid chromatography-mass spectrometry from 484 pancreatic cancer patients. Isocitrate and aconitate in the tricarboxylic acid cycle were statistically significantly associated with survival outcomes. Hazard ratios for death of 1.89 for isocitrate (95% CI 1.06–3.35, p < 0.001) and 2.54 for aconitate (95% CI 1.42–4.54, p < 0.001) were suggested. Moreover, Moore *et al*.^34^ reported enhanced metabolomics analysis identified metabolic pathways that may assist in differentiating pancreatic cancer stages that do not occur in a linear stepwise progression. Among the 215 measured plasma metabolites, five principal metabolic components were identified as exhibiting strong correlation with disease burden in pancreatic cancer. Specifically, pancreatic neuroendocrine tumor was associated with high uric acid, methionine, intraductal papillary mucinous neoplasm with high amino acids, locally advanced pancreatic cancer with both high fatty acids and high polyamines, and metastatic pancreatic ductal adenocarcinoma with high tricarboxylic acid cycle, while local pancreatic cancer showed no predominance of specific principal components.

Metabolite pathways are still uncertain. Many metabolites are currently being studied in pancreatic cancer, as there are many pathways to explain them. According to a recent review, alanine aspartate and glutamate metabolism, glycine serine and threonine metabolism, and taurine and hypotaurine metabolism are the three most prominent pathways^35^. From the study, a total of 132 potential metabolite-based biomarker candidates were selected. Among them, amino acids were the dominant biomarkers. Seven other pathways were also enriched, including arginine and prolene metabolism; aminoacryl-tRNA biosynthesis; methane metabolism; valine, leucine, and isoleucine biosynthesis; nitrogen metabolism; cyanoamino acid metabolism; and synthesis and degradation of ketone bodies.

In the present study, we successfully developed a preoperative serum metabolome-based nomogram with which to predict 1-year disease-free survival probability in patients with resected pancreatic cancer. Only preoperatively detectable parameters, including CA 19–9 and three PC derivatives (PC.aa.C38_4, PC.ae.C42_5, and PC.ae.C38_6), are needed. The accuracy and model performance were found to be acceptable. In clinical settings, potential candidates for surgical resection of resectable pancreatic cancer comprise the target clinical population for the application of this nomogram. If the values of preoperative serum CA 19–9 and the three PC derivatives are known, 6-month and 1-year disease-free survival probability after pancreatectomy can be easily estimated using the nomogram, even before surgery. Therefore, patients and their families will be able to gain additional information on the potential prognostic benefit of surgical resection during the preoperative decision-making process for radical pancreatectomy. Surgeons and medical oncologists will be able to individualize the follow-up strategy according to the estimated disease-free survival probability after radical pancreatectomy. We are providing free on-line access to this nomogram that we developed (http://103.22.220.149:8080/service/kang/home2.jsp).

Recently, the potential oncological role of neoadjuvant treatment followed by surgery has been actively investigated in advanced pancreatic cancer^36,37^. Several studies on the clinical application of neoadjuvant treatment in resectable pancreatic cancer showed no oncological benefit of neoadjuvant treatment in resectable pancreatic cancer^38^. However, if the present nomogram estimated poor 6-month or 1-year survival following surgical resection (for example, less than 50%), the initial planned treatment strategy of surgical resection may be changed to include neoadjuvant treatment before surgical resection, even in patients with resectable pancreatic cancer, which redefines the concept of a patient-oriented surgical approach to “resectable” pancreatic cancer. The clinical value should be validated with well-designed prospective randomized control studies in the near future.

In order to eliminate selection bias, survival analysis was conducted by a statistician, not the researchers who performed the metabolite analysis, without knowing which patient’s preoperative metabolites was enrolled. However, several limitations should be considered when interpreting the present results: This study was retrospective in nature, with a limited number of patients. The current nomogram was established based on long-term oncological outcomes (recurrence) of patients with surgical resection; however, the preoperative clinical application of this nomogram is based on the assumption that patients had already undergone pancreatectomy. Thus, it can be said that this study is limited to a “proof-of-concept.” However, the fact that a retrospective study involving a small number of patients can be overcome by the development of a practical nomogram based on clinical data like this study. Because of these reasons, the performance of this model may need to be re-validated in a large-volume prospective study.

In summary, patients with resected pancreatic cancer can be categorized according to their different metabolomes in order to predict early cancer recurrence. Preoperative detectable parameters, including serum CA 19–9 and the three PC derivatives PC.aa.C38_4, PC.ae.C42_5, and PC.ae.C38_6 were used to develop a model for preoperatively predicting early tumor recurrence in patients who underwent pancreatectomy for pancreatic cancer. The specific roles of the three individual PC derivatives also need to be carefully reevaluated. This model will be helpful in decision-making for surgery and establishing follow-up strategies for patients with resected pancreatic cancer. Further study is mandatory.

## Supplementary information

Dataset 1

Dataset 2

Supplementary 3 - Table
