# Supplementary material for: Developing a preoperative serum metabolome-based recurrence-predicting nomogram for patients with resected pancreatic ductal adenocarcinoma
Source: Sci Rep. 2019 Dec 9;9:18634. doi: 10.1038/s41598-019-55016-x (PMC6901525; doi:10.1038/s41598-019-55016-x)
Supplement: Supplementary file 3 — Supplementary 3 - Table [file 41598_2019_55016_MOESM3_ESM.docx]

**Developing a preoperative serum metabolome-based recurrence-predicting nomogram for patients with resected pancreatic ductal adenocarcinoma**

­­­­­Seoung Yoon Rho^1,2^, Sang-Guk Lee^3^, Minsu Park^4^, Jinae Lee^4^, Sung Hwan Lee ^1,5^, Ho Kyoung Hwang^1,2^, Min Jung Lee^6^, Young-Ki Paik^6^, Woo Jung Lee^1,2^, Chang Moo Kang^1,2^

^1^Division of Hepatobiliary and Pancreatic Surgery, Department of Surgery, Yonsei University College of Medicine, Seoul, Korea

^2^Yonsei Pancreatobiliary Cancer Center, Severance Hospital, Seoul, Korea

^3^Department of Laboratory Medicine, Severance Hospital, Yonsei University College of Medicine, Seoul, Korea

^4^Biostatistics Collaboration Unit, Yonsei University College of Medicine, Seoul, Republic of Korea

^5^Department of Systems Biology, University of Texas MD Anderson Cancer Center

^6^Yonsei Proteome Research Center and ‡Department of Integrated OMICS for Biomedical Science and Department of Biochemistry, College of Life Science and Biotechnology, Yonsei University

**Supplementary 3-1. LC parameters used for metabolites measurement**

|  | Time (min) | Flow rate (mL/min) | Mobile phase A (%) |
| --- | --- | --- | --- |
| LC-MS/MS | 0.0 | 0.5 | 100 |
|  | 0.5 | 0.5 | 100 |
|  | 5.5 | 0.5 | 5 |
|  | 6.5 | 0.5 | 5 |
|  | 7.0 | 0.5 | 100 |
|  | 9.5 | 0.5 | 100 |
| FIA-MS/MS | 0.0 | 0.03 | MS mobile phase prepared by Biocrates Solvent Ⅰ in isocratic mode |
|  | 1.6 | 0.03 |  |
|  | 2.4 | 0.2 |  |
|  | 2.8 | 0.2 |  |
|  | 3.0 | 0.03 |  |

LC-MS/MS, liquid chromatography-tandem mass spectrometry; FIA, flow injection analysis.

**Supplementary 3-2. MS parameters used for metabolites measurement**

| Instrument | Parameter | LC‐MS/MS | FIA-MS/MS | |
| --- | --- | --- | --- | --- |
|  |  |  | Positive mode | Negative mode |
| QTRAP 5500 | CUR (psi) | 20 | 20 | 20 |
|  | IS (V) | 5500 | 5500 | -4500 |
|  | TEM (°C) | 500 | 200 | 200 |
|  | GS1 (psi) | 40 | 40 | 40 |
|  | GS2 (psi) | 50 | 50 | 50 |
|  | CAD (psi) | 6 | 6 | 6 |
|  | EP (V) | 10 | 10 | 10 |

CUR, curtain gas; IS, ion spray voltage; TEM, temperature; GS1/GS2, ion source gas 1 and 2; CAD, collisionally activated dissociation gas; EP, entrance potential; LC-MS/MS, liquid chromatography-tandem mass spectrometry; FIA, flow injection analysis.
